# Marine-Derived Compounds Combined with Nanoparticles: A Focus on the Biomedical and Pharmaceutical Sector

**DOI:** 10.3390/md23050207

**Published:** 2025-05-13

**Authors:** Laura M. Teixeira, Catarina P. Reis, Rita Pacheco

**Affiliations:** 1 Departamento de Química e Bioquímica, Faculdade de Ciências, Universidade de Lisboa, 1749-016 Lisboa, Portugal; fc57476@alunos.ciencias.ulisboa.pt; 2 Centro de Química Estrutural, Institute of Molecular Sciences, Faculdade de Ciências, Universidade de Lisboa, 1749-016 Lisboa, Portugal; 3 Institute for Medicines (iMed.ULisboa), Faculdade de Farmácia, Universidade de Lisboa, 1649-003 Lisboa, Portugal; 4 Instituto de Biofísica e Engenharia Biomédica (IBEB), Faculdade de Ciências, Universidade de Lisboa, 1749-016 Lisboa, Portugal; 5 Departamento de Engenharia Química, Instituto Superior de Engenharia de Lisboa, 1959-007 Lisboa, Portugal

**Keywords:** marine bioactive compounds, nanoparticles, green synthesis, marine polysaccharides, drug delivery systems

## Abstract

The ocean is an extraordinary natural source of a wide range of bioactive compounds. These compounds, including proteins, phenolics, polysaccharides, pigments, vitamins, and fatty acids, possess unique biological properties that are increasingly being explored in the field of nanotechnology across diverse sectors. Among marine-derived nanoparticles, promising applications have emerged in the biomedical and pharmaceutical fields, particularly metallic nanoparticles and polysaccharide-based drug delivery systems. This review provides a unique perspective on the integration of two research areas: the exploration of marine bioresources as bioactive compounds sources with nanotechnological methodologies to develop sustainable, safe, stable and functional marine-derived NPs. It highlights recent advancements in the green synthesis of MNPs and the formulation of drug delivery systems using marine polysaccharides. This review also describes the recent trends over the past ten years and discusses the major challenges and limitations associated with these approaches, including variability in biological sources, batch-to-batch inconsistency, mechanistic uncertainties, and difficulties in reproducibility and scalability. Furthermore, it emphasizes the need for standardized protocols and the integration of life cycle assessments (LCA) to evaluate environmental and economic viability for effective translating marine-derives nanoparticles from research to clinical applications.

## 1. Introduction

The ocean, which covers around 70% of the Earth’s surface, is the most extensive ecosystem on the planet, supporting about 90% of all living organisms [1,2]. This vast and complex aquatic world remains largely unexplored, yet it supports a rich variety of life and remarkable biological diversity [3,4].

Marine organisms, such as microorganisms, microalgae, macroalgae (seaweeds), sponges and crustaceans (crabs, shrimps, crayfish and lobsters) are known to be sources of a diverse array of compounds, each with a unique chemical structure and properties [5,6,7]. To date, approximately 36,000 compounds have been successfully isolated from marine organisms [6], including proteins [8,9], pigments [10,11,12], polysaccharides [13,14,15], and phenolics [16,17], among others. When the bioactive compounds of marine origin were compared with those of terrestrial life, the former demonstrated higher bioactivity [18]. Therefore, the ocean is regarded as one of the most important sources of natural bioactive compounds [19].

Several marine bioactive compounds have demonstrated a wide range of biological properties, from antioxidant [20,21], anti-inflammatory [22], anti-coagulant [23,24], anti-cancer [25,26], to anti-diabetic activities [27], as well as cardioprotective effects [28]. Moreover, these compounds are biodegradable, biocompatible, non-toxic and have a low cost, making them highly appealing for research [29]. Therefore, marine bioactive compounds have been applied in countless areas, namely in medicine, food and dietary supplements, and cosmetics [2,7].

Recently, marine bioactive compounds have been extensively applied in nanotechnology [30]. Nanotechnology is a field of research focused on the manipulation of materials at the nanoscale [31]. Among the various nanomaterials, nanoparticles (NPs), with diameters normally ranging from 1 to 1000 nm, have been widely studied due to their unique chemical, physical and biological properties [32,33]. These properties have enabled NPs to be applied in various industries, including pharmaceuticals, cosmetics, textiles, electronics, as well as in agriculture and environmental remediation [34,35].

In the agricultural sector, these NPs serve as nanofertilizers and biopesticides, enhancing plant growth and reducing dependence on harmful agrochemicals [36]. For instance, silver NPs are well known for their potent anti-microbial activity and have been effectively used to control agricultural pathogens [37]. In environmental contexts, NPs of marine origin have shown significant potential in wastewater treatment, particularly in the remediation of organic pollutants [38]. Additionally, microalgae-derived nanomaterials, such as cobalt, have demonstrated catalytic properties for environmental purification, by being able to oxide CO [39]. In the food industry, marine-based NPs are explored as preservatives and for enhancing nutritional value [40,41,42]. Cosmetics benefits from NPs through improved skin penetration and ingredient delivery, such as in sunscreens [43,44]. In textiles, NPs offer antimicrobial effects, UV protection, water resistance, and support the development of smart fabrics [45]. Electronics utilize nanocrystals for their electrical and optical properties, enabling cost-effective, large-scale device fabrication, such as biosensors [46]. Overall, their diverse bioactive properties highlight the multifunctional role of marine-derived NPs in various industrial fields.

Among these applications, the medical field represents a significant growth sector. The high interest in NPs is a result of their numerous advantages, including bioavailability, biostability, biodistribution, and their ability to overcome biological barriers [47,48,49]. NPs have been used in diagnostics and as biomarkers and have also revolutionized drug delivery systems (DDS), which are designed for transport and release therapeutics agents into the human body [34,50,51]. By enabling targeted delivery and controlled release, as NPs improve therapeutic efficacy while reducing side effects in drug administration [50,51].

Regarding NP synthesis, several methods have been employed, including chemical, physical and biological approaches [52]. Recently, the synthesis of NPs, more specifically metallic NPs (MNPs), has been achieved through the application of natural products from fungi, bacteria, plants and algae. This type of approach, known as green synthesis, is a simple, inexpensive and environmentally friendly method, as it does not produce toxic by-products [30,53].

Marine bioactive compounds have shown great potential not only in the green synthesis of NPs but also as DDS. In particular, marine-derived polysaccharides, have been extensively studied as DDS due to their biocompatibility, biodegradability, and ability to form various nanostructures suitable for drug encapsulation and controlled release [29].

This review provides an overview of the latest advances in the application of marine-based compounds in nanotechnology, with a particular focus on the biomedical and pharmaceutical sectors. Specifically, it explores the potential of microalgae and macroalgae in the synthesis of MNPs, as well as the current challenges associated with this synthesis method. Additionally, the review evaluates the potential of polysaccharides from various marine sources, namely fucoidan, chitosan and alginate, for use in DDS. The integration of marine bioactive compounds in both MNP synthesis and DDS development highlights their significant role in advancing nanomedicine innovation. This approach not only supports sustainable and eco-friendly NP production but also contributes to more efficient and safer drug delivery solutions.

## 2. Methods

This review compiles articles obtained through a search on Google Scholar and PubChem databases using the keywords “marine bioactive compounds”, “algae”, “microalgae”, “macroalgae”, “crustaceans”, “nanoparticles”, “metallic nanoparticles”, “gold nanoparticles”, “silver nanoparticles”, “polysaccharides”, “fucoidan”, “chitosan”, “alginate”, “drug delivery system”, ”ecological impact”, either alone or in combination. Only articles published in English were selected, with preference given to those published in the last 10 years.

## 3. Marine Bioactive Compounds Sources

**Figure 1:**
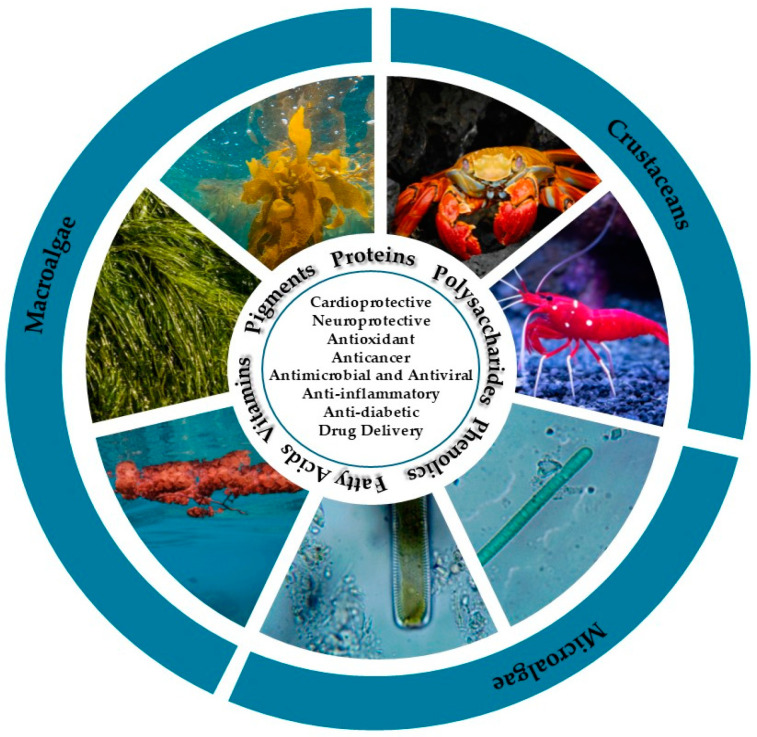
Marine bioactive compound sources and their biomedical and pharmaceutical application.

Marine organisms have various physiological and biochemical mechanisms that are essential for their communication, reproduction and defense. These complex processes ultimately result in the production of a wide range of bioactive compounds with unique chemical structures and biological activities that have broad biomedical and industrial applications [54]. Among these, macroalgae and microalgae are particularly recognized for producing a variety of proteins, polysaccharides, phenolics, pigments, vitamins, and essential fatty acids, whereas marine crustaceans are major sources structural biopolymers, such as chitin and chitosan [29,55]. An overview of marine-derived bioactive compounds, their sources, and key biomedical and pharmaceutical applications is presented in Figure 1.

In recent years, the global market for marine-derived drugs has expanded significantly; by 2024, this market was valued at USD 4.18 billion, and it is expected to reach USD 9.28 billion by 2034, with a compound annual growth rate (CAGR) of 8.3% [56].

This market growth reflects the ongoing exploration of marine biodiversity and the successful development and approval of several marine-based drugs. Examples of marine drugs currently in clinical use and available on the market include vidarabine, brentuximab vedotin and enfortumab vedotin, demonstrating the successful translation of marine bioactive compounds into therapeutic products [57]. Similarly, the market for algae-based products is also experiencing significant growth, and it is expected to reach USD 3.86 billion in 2025, increasing to USD 5.52 billion in 2030, with a CAGR of 7.44% [58].

The growth of these markets is attributed to an increase in consumer awareness of the health benefits of marine-derived bioactive compounds. In addition, these compounds are generally perceived as safe and are therefore culturally accepted [59]. As the demand for these compounds increases, companies are investing more in innovative production methods and extraction techniques, in order to enhance the yield and bioavailability of the bioactive compounds [60].

### 3.1. Microalgae Sources

Microalgae are single-cell, photosynthetic microorganisms that usually inhabit aqueous environments, such as marine systems and freshwaters [52,61,62]. These organisms are generally classified as prokaryotes or eukaryotes but can also be classified according to a variety of features, such as the organization of their photosynthetic membranes, pigmentation, and other morphological characteristics. It is currently estimated that there are more than 50,000 species of microalgae, of which about 30,000 have been investigated, demonstrating the high biodiversity of microalgae [52,63]. The most abundant classes of microalgae are *Chlorophyceae*, *Bacillariophyceae* and *Cyanophyceae* [62,63].

Microalgae produce a vast range of bioactive compounds, the content of which varies between strains [52]. For example, *Chlorella* sp. is recognized for being a rich source of polyunsaturated fatty acids (PUFAs), and pigments, such as chlorophyll and carotenoids, which contribute to its antioxidant properties [64,65]. Similarly, *Dunaliella salina* is known to produce a high content of pigments, namely β-carotene, a powerful antioxidant with applications in the cosmetic industry [66,67]. Another widely studied microalgae is *Spirulina platensis*, recognized for its content of carbohydrates, phenolic compounds and vitamins, making it a valuable ingredient in dietary supplements [68].

Due to their rich biochemical composition, microalgae are considered a promising and sustainable natural source of many bioactive compounds with a wide range of applications [52].

In the cosmetic industry, microalgae-derived compounds, such as pigments, are widely used in skincare formulations for their anti-aging and UV-protection properties [62]. For example, the pigment astaxanthin from *Haematococcus pluvialis*, has been extensively studied for its ability to reduce oxidative stress and improve skin, making it a key ingredient in anti-aging creams and sunscreens [69]. Another example is scytonemin, a UV-absorbing pigment produced by *Nostoc* sp. and *Chlorogloeopsis* sp., which is widely used in sunscreens and other photoprotective formulations due to its ability to block UV-visible radiation [70]. In food and dietary supplements, microalgae are valued for their high protein, vitamin, mineral, and essential fatty acid content. For example, *Chlorella* and *Spirulina* are rich in iron, calcium, and zinc, and studies have shown that their consumption can enhance iron absorption and help combat anemia, as demonstrated in anemic rats [71]. Furthermore, PUFAs such as docosahexaenoic acid (DHA) and eicosapentaenoic acid (EPA) from *Schizochytrium* and *Nannochloropsis* have been incorporated into functional foods and supplements due to their well-documented benefits for cardiovascular and cognitive health [72,73]. In the pharmaceutical sector, bioactive compounds from microalgae have been investigated for their anti-inflammatory, antimicrobial, and anticancer properties. For instance, phycocyanin, a blue pigment from *Spirulina*, has shown promising anti-inflammatory and antioxidant effects, making it a potential candidate for drug development [74,75].

### 3.2. Macroalgae Sources

Macroalgae, commonly referred to as seaweed, are photosynthetic organisms that play a crucial role in the aquatic ecosystems [18]. Currently, around 9000 species of macroalgae are known, exhibiting various colors. As a result, seaweeds are often classified based on their pigmentation and morphological characteristics [18,76]. These organisms are typically categorized into three main groups: brown algae (*Phaeophyceae*), red algae (*Rhodophyta*) and green algae (*Chlorophyta*) [77].

Brown algae are very diverse, and the majority of marine algae are found in the ocean. Their pigmentation ranges from yellow to dark brown, and in terms of their shape and size, brown algae can be small and filamentous or part of a large group with a complex structure [77,78]. Red algae are the most primitive algae and are the second largest group of marine algae. When compared to the other types of algae, red algae are found in more diverse environments [77,79]. Green algae present a dark yellow to dark green color, and are found in smaller quantities than brown and red algae and [77].

Macroalgae have been widely used in various industries, as they are a source of various biodegradable and non-toxic bioactive compounds. Macroalgae such as *Gracilaria edulis* and *Gracilaria corticata* are known for their content in proteins, lipids and carbohydrates, being recently used in the food and dietary supplements industries for their nutritional benefits [80]. Another example is *Ulva rigida*, which is rich in phenolic compounds, vitamins, and pigments, and has been extensively studied for its antioxidant properties [81]. A brown alga which is extensively studied is *Fucus vesiculosus*, due to its content in phenolic and other bioactive compounds, with applications in the medical field, particularly its potential for managing conditions like hypercholesterolemia [82,83]. Moreover, *Fucus vesiculosus* is notable for its ability to synthesize fucoidan, a polysaccharide with promising antioxidant, and anti-inflammatory activities [84,85].

Given the various bioactive compounds that macroalgae present, their demand has increased, resulting in their cultivation and utilization across various sectors, including pharmaceuticals, cosmetics, and supplements [86]. For example, in the pharmaceutical industry, *Gracilariopsis lemaneiformis* peptides, such as FQIN [M(O)] CILR and TGAPCR, have demonstrated significant anti-hypertensive effects in hypertensive rat models, highlighting their therapeutic potential for blood pressure management [87]. In the food supplement industry, SeapolynolTM, a supplement derived from seanol, a phenolic compound from *Ecklonia cava*, has been approved by the European Food Safety Authority for its anti-diabetic properties, showing promising results in controlling blood sugar levels [88]. In the cosmetic sector, fucoidan derived from *Laminaria japonica* is utilized for its skin-whitening properties, enhancing the market potential of macroalgae in skincare and anti-aging products [89].

### 3.3. Crustacean Sources

Crustaceans, including crabs, shrimps, lobsters and crayfish, are typically classified according to their habitat, which can be marine, freshwater or terrestrial environments [90], exploited mostly by the food industry [18,90,91]. However, these industries often produce a significant amount of waste, as by-products like shells, heads, and exoskeletons are commonly discarded. These by-products can account for 50–70% of the total weight of the crustaceans, resulting in an estimated annual production of 6–8 million tons of waste [91,92]. Despite being discarded, crustacean by-products are rich in bioactive compounds that hold considerable potential for several applications, including in the biomedical, pharmaceutical, food, and cosmetic industries [93]. This has led to a growing interest in utilizing these by-products as sustainable alternatives to synthetic materials, contributing to more eco-friendly solutions across various sectors.

Approximately 15–40% of the waste from these crustaceans consists of chitin and its derivatives, which have been applied in a variety of industries [91]. In addition to chitin and its derivatives, such as chitosan, crustacean-derived polysaccharides include sulphated polysaccharides, acidic polysaccharides and chitooligosaccharides [92]. These compounds are increasingly being studied for their numerous functional and biological properties. Such properties make them promising candidates for a wide range of applications across several areas including the pharmaceutical and cosmetic sectors [91].

In the pharmaceutical sector, chitosan has been widely used as a weight management supplement due to its ability to regulate fat absorption in the body [94]. Furthermore, chitosan extracted from the exoskeleton of freshwater crab *Potamonautes miloticus* has demonstrated wound-healing properties in albino rats, highlighting its potential for tissue regeneration and wound care [95]. Additionally, sulphated polysaccharides derived from *Bellamya quadrata*, have been shown to enhance the stability of atherosclerotic plaque, suggesting their promising role in cardiovascular treatment [96]. In the cosmetic sector, chitosan and other polysaccharides are incorporated into skincare formulations for their anti-aging and skin-improving properties. These ingredients are valued for their ability to form films that improve skin elasticity, retain moisture, and protects against UV radiation. There are various chitosan-based products for cosmetic use that are commercially available, some examples are Curasan™, Hydamer™ and Zenvivo™, which are applied in creams, lotions, and ointments [97]. In addition to their wellness and therapeutic properties, marine-derived bioactive compounds from crustaceans have also been applied in the food sector. Chitosan and chitin derivatives are commonly used as natural preservatives due to their antimicrobial properties, extending the shelf life of food products without the use of synthetic chemicals [98] For example, chitosan was verified to reduce the growth of a fungus found in fruits, *Botryosphaeria* sp., being ideal for fruit preservation [99].

The following sections will review recent trends in the use of nanotechnology for the development of marine-derived drugs, focusing on the use and incorporation of the above-mentioned bioactive compounds into NP formulations. Particular attention will be given to the green synthesis of NPs and to their biomedical and pharmaceutical applications, highlighting their potential for innovative medical solutions and advanced drug delivery systems. Advances in nanotechnology-based approaches have been shown to improve bioavailability, optimize distribution, and increase therapeutic efficacy, leading to a more effective and targeted use of marine-derived bioactive compounds [100].

## 4. Metallic Nanoparticles from Marine Sources

Among the various nanotechnology-based approaches, MNPs have attracted significant attention due to their unique physicochemical properties, making them valuable for a wide range of biomedical and pharmaceutical applications. MNPs have unique physicochemical properties, such as high surface area, biological and physicochemical properties [101,102]. MNPs can be tailored by modifications to their surface chemistry, charge, size and morphology, allowing precise control of their behavior, enhancing their potential in drug delivery, diagnostics, and imaging [102,103].

### 4.1. Metallic Nanoparticles Synthesis

Two main approaches, the top–down and bottom–up, are commonly applied for the synthesis of MNPs. The top–down approach involves the reduction of bulk materials into nanoscale structures using physical techniques such as mechanical milling, laser ablation, sputtering, or other chemical methods such as electrochemical reduction, and chemical exfoliation [32,47,104]. These techniques typically require substantial energy input, in which during the reduction of size, the high pressure and temperature may result in NPs with lower uniformity, affecting the chemical and physical properties of the MNPs [105].

In contrast, the bottom–up approach involves the assembly of atomic or molecular species into an organized nanostructure. Common chemical methods include precipitation, spinning, chemical vapor deposition (CVD) and chemical reduction [47,104]. In addition, biological methods, often referred to as green synthesis, have emerged as eco-friendly and sustainable alternatives. These methods use natural products such as plant extracts, bacteria, fungi, and algae, as reducing and stabilizing agents for NPs formation [32,106,107]. This approach offers enhanced control over particle size and composition and is generally considered more cost-effective [32,104]. Unlike chemical synthesis, which often requires toxic reagents and synthetic stabilizers, or physical methods, which are energy-intensive and costly, green synthesis provides a safer, more cost-effective, and environmentally friendly route to MNPs production [32].

Following synthesis, the characterization of MNPs is essential to confirm their size, morphology, and structural integrity. Several analytical techniques are employed to assess these properties. Transmission electron microscopy (TEM), scanning electron microscopy (SEM) and atomic force microscopy (AFM) are widely used to visualize NPs morphology and size, and evaluate aggregation. These techniques provide high-resolution imaging at the nanoscale, with TEM providing atomic-level detail and AFM enabling 3D surface profiling with nanometer precision [47,108].

Other techniques, such as dynamic light scattering (DLS), a rapid and non-invasive approach, is used to determine the hydrodynamic size distribution, polydispersity index (PdI) and the surface change of NPs in a suspension. Additionally, X-ray diffraction (XRD) is employed to assess the crystalline nature of the NPs, while Fourier-transform infrared spectroscopy (FTIR) identifies functional groups, and the nature of the compounds present in the NPs [47,108]. UV-visible (UV-vis) spectroscopy is also widely applied providing quantitative information on the optical properties of NPs, including insights into how metal ion concentration during synthesis influences the stability of MNPs [105,108]. Taken together, these characterization methods provide essential insight into the physicochemical properties of MNPs, supporting the optimization of the synthesis methodologies for specific applications [108].

### 4.2. Green Synthesis of Metallic Nanoparticles

Algae, in particular, have been extensively used in the green synthesis of MNPs due to their sustainability and eco-friendly nature. Their rich content of bioactive compounds, such as polysaccharides, proteins, phenolics, and pigments, not only facilitates the reduction and stabilization of metal ions but also provides additional functional properties to the resultant MNPs [106,107]. The natural metabolites play a crucial role in influencing the size, morphology and physicochemical properties of the resulting NPs, and can therefore be selected to obtain the desired properties for specific applications [109].

**Figure 2:**
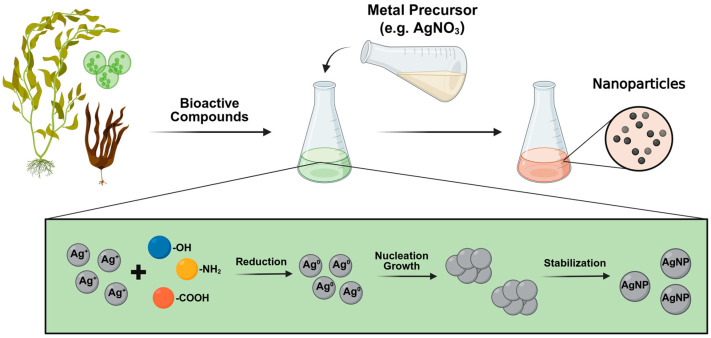
Green synthesis of metallic nanoparticles (MNPs) using algae. Algae biomass or extract is combined with silver nitrate (AgNO_3_) precursor solution. Algae metabolites, rich in hydroxyl (-OH), amino (-NH_2_), and carboxyl (-COOH) groups, reduce silver ions (Ag^+^) to neutral atoms (Ag^0^), initiating nucleation. The nanoparticles grow and stabilize. The formation of NPs is indicated by a visible color change in the solution.

The green synthesis process begins by combining the algae with a metallic precursor (Figure 2). The synthesis proceeds through three main phases. In the activation phase, algae metabolites, which are rich in key functional chemical groups such as hydroxyl (-OH), amine (-NH_2_), and carboxyl (-COOH) groups play a crucial role in reducing the metal ions in the precursor solution to neutral atoms, initiating the nucleation process, usually indicated by a visible color change in the solution [110]. Subsequently, during the growth phase, the MNPs begin to aggregate and increase in size [47,52,53]. During this phase, the stability of the NPs depends on their interaction with the bioactive compounds, which act as both capping and stabilizing agents [111]. Finally, in the termination phase, the MNPs reach thermodynamic stability. The morphology and size achieved depend on the composition and the concentration of the source algal metabolites used [47,52,53].

Various molecules found in algae have been reported for their role in the green synthesis of MNPs. These include proteins, polysaccharides, pigments and phenolic compounds, particularly those containing functional groups such as hydroxyl, amine and carboxyl groups, which act in the reduction of the metal precursor and as capping and stabilizing agents [110,111].

For instance, Venkatesan et al. reported the synthesis of silver NPs (AgNPs) using an aqueous extract of *Ecklonia cava*. The synthesized AgNPs, with a size range of 15–30 nm and a spherical morphology, were characterized using DLS and TEM analysis, respectively. FTIR analysis revealed that the phenolic compounds in the extract were responsible for both reducing the precursor and capping the AgNPs, ensuring their stability [112].

Polysaccharides, including alginate, fucoidan and laminarin, extracted from *Saccharina cichorioides* and *Fucus evanescens*, have also been identified as effective reducing and stabilizing agents in NPs synthesis. Yugay et al. also demonstrated that these polysaccharides facilitated the formations of spherical AgNPs, with a size range from 45 to 64 nm confirmed by TEM and DLS analysis. Among these polysaccharides, alginate showed the highest catalytic activity [113].

Additionally, Araya-Castro et al. reported the synthesis of copper NPs (CuNPs) using proteins isolated from the macroalgae *Macrocystis pyrifera*. DLS and TEM confirmed sizes ranging from 2 to 50 nm and spherical morphology, were confirmed by both DLS and TEM analyses. FTIR analysis of the proteins indicated that cysteine was primarily responsible for both the reduction and stabilization of the CuNPs [110]. Although individual biomolecules were reported to have reducing and capping capabilities in the synthesis of MNPs, most of the studies use whole algae or algal extracts as the source of these functional components, rather than purified or isolated molecules.

In the green synthesis of MNPs using algae, two primary biosynthetic pathways have been identified: intracellular and extract-based synthesis. These pathways differ on where the MNPs formation occurs: the intracellular route, where NPs are produced inside the algae cells, and the extract-based synthesis occurring outside the cells using the extracted algae metabolites incubated with the metal precursor [47,109].

Intracellular synthesis of MNPs is a method that does not require any pre-treatment of the algae, and it is carried out inside the algae cells [114]. The potential of using algae in the synthesis of MNPs results from their remarkable tolerance to environmental stressors, including exposure to heavy metal ions. Algae, known for their ability to hyperaccumulate these ions, can reduce these metals, leading to the production of a range of valuable MNPs [47,106,115].

In this process, when algal cells are exposed to a metal precursor solution, positively charged metal ions are transported across the cell membrane. Inside the cell, various biomolecules, including enzymes, pigments, carbohydrates, and other reducing agents, facilitate the reduction of metal ions to their neutral (zero-valent) state. These atoms then nucleate and aggregate into MNPs, whose size and morphology can vary depending on the algal species and intracellular conditions [111].

Several studies have successfully employed the intracellular synthesis route using different algal species and metal precursors. For instance, in a study by Sicard et al., AuNPs were synthesized by mixing chloroauric acid (HAuCl) with the microalgae *Klebsormidium flaccidum*, which was cultivated under controlled temperature and luminosity conditions. During the intracellular synthesis process, the microalgae were encapsulated in a silica gel. The formation of AuNPs was confirmed through optical and electron microscopy, where a color change in the chloroplasts from green to purple indicated the reduction of gold salts by the algae. The AuNPs were characterized by UV-visible spectroscopy, where AuNPs showed their characteristic absorption peaks. Additionally, TEM analysis revealed that the AuNPs had a particle size ranging from 10 to 15 nm in diameter. An interesting finding in this study was that the silica gel protected the microalgae from the toxic effects of the gold [116].

Similarly, a green macroalgae, *Rhizoclonium fontinales*, was selected by Parial et al. for the intracellular synthesis of AuNPs, this time using hydrogen tetrachloroaurate salt (HAuCl_4_ ·*X*H_2_O). The formation of the AuNPs was confirmed by the characteristic absorption peak of gold in the UV-vis spectrum. AuNPs were formulated at different pH values (5, 7 and 9), and characterized by high resolution TEM (HRTEM) and DLS. In this study, it was found that increasing pH decreases both particle size and PdI. Additionally, it was observed that the morphology of AuNPs changed according to the pH value. At a pH 5, the AuNPs presented various morphologies, such as triangles, hexagons and rods, whereas at pH 7 and 9, they predominantly displayed a spherical shape. The effects of the gold exposure to the macroalgae were also evaluated, demonstrating a decrease of the algae’s content of chlorophyll, carbohydrates and proteins and the upregulation of some stress-related enzymes [117].

The extract-based synthesis of MNPs is a process that occurs after the extraction of metabolites, pigments, enzymes, among others, from the algae biomass. To achieve this, the algae needs to be pre-treated, washed and blended to obtain an extract. The extract is then combined with the metal precursor to synthesize the MNPs [47,52]. Compared to the intracellular process, the extract-based process is often considered more practical and scalable due to easier downstream processing of NPs. In addition, it is possible to evaluate the effect of different types of extracts by changing certain processing steps, such as temperature, pH or extraction solvents [52].

In a study by Muthusamy et al., AgNPs were synthesized by combining silver nitrate (AgNO_3_) with an extract of *Spirulina platensis*. To prepare the extract, the microalgae was washed, dried and ground to obtain a powder. AgNPs were formulated at different pH values, (5, 6, 7, and 8), with the formation of the NPs indicated by a color change to yellow. The presence of AgNPs was confirmed by the presence of their characteristic absorption peak in the UV-vis spectrum, as further validated by FTIR. TEM analysis revealed that the AgNPs had a spherical shape with particle size ranging from 5 to 50 nm. Additionally, the study concluded that at higher concentrations, AgNPs exhibited antibacterial activity, particularly against *Staphylococcus* sp. and *Klebsiella* sp. [118].

González-Ballesteros et al. synthesize AuNPs using an extract from the brown macroalgae *Cystoseira baccata*, combined with HAuCl. The extract was prepared by cutting the algae into pieces and boiling them. The formation of the AuNPs was confirmed by their characteristic peak in the UV-vis spectrum. TEM images revealed an average particle size of 8.4 nm of the AuNPs. The cytotoxicity of the synthesized AuNPs was evaluated against two human colon cancer cell lines (Caco-2 and HT-29) and a neonatal dermal fibroblast cell line (PCS-201-010). The results showed that the AuNPs exhibited toxicity targeted against the cancer cell lines but had no toxic effects on fibroblasts. Furthermore, the study concluded that the AuNPs induced apoptosis in cancer cells through both the mitochondrial and extrinsic pathways [119].

In a study by Kathiraven et al., AgNPs were synthesized using an extract from green macroalgae *Caulerpa racemosa*. The macroalga was washed, sliced, dried, and ground into a powder to prepare the extract. The extract was combined with the metal precursor, AgNO_3_, and the formulation of the AgNPs was confirmed by UV–vis and FTIR spectroscopy. TEM analysis demonstrated that the AgNPs had spherical and triangular morphologies, with an average particle size ranging from 5 to 25 nm. Additionally, the synthesized AgNPs demonstrated antibacterial properties against two human pathogens, namely *Proteus mirabilis* and *Staphylococcus aureus* [120].

This extract-based synthesis method has also been applied beyond the biomedical and pharmaceutical sector, particularly in environmental remediation. AgNPs synthesized using extracts from *Spirulina platensis*, *Chlorella vulgaris*, and other microalgae have demonstrated considerable potential in water treatment. These biologically derived AgNPs have been shown to effectively degrade synthetic dyes and organic pollutants [121,122].

Although green synthesis methods of MNPs are relatively simple, it is reported that several factors can significantly influence their characteristics. Understanding these factors is essential for optimizing the synthesis process and adapting the properties of the NPs to specific biomedical, environmental and industrial applications [123].

### 4.3. Parameters Affecting the Green Synthesis of Metallic Nanoparticles

The green synthesis of MNPs has proven to be very promising. However, this process is influenced by some factors such as temperature, pH, metallic precursor concentration, algae type and concentration, among others [123]. These are considered critical factors, as they can influence the synthesis and, consequently, the characteristics of the MNPs obtained [124].

#### 4.3.1. Temperature Effect

Temperature is a critical parameter influencing the green synthesis of MNPs, though its effects can vary depending on the biological source and synthesis conditions. For example, Aboelfetoh et al., formulated AgNPs using an extract of a green macroalgae, *Caulerpa serrulata*, at two temperatures: 27 °C and 95 °C. TEM images showed that higher temperatures led to the formation of smaller NPs with a spherical shape and an average particle size of 10 nm. This was attributed to accelerated reactants consumption at elevated temperatures, leading to rapid nucleation and smaller particle formation [125]. Conversely, Yilmaz Öztürk et al. formulated AgNPs using an extract of a red macroalgae, *Gelidium corneum*, at different temperatures (60 °C, 70 °C, 80 °C and 90 °C) and the optimal temperature was determined to be 80 °C, presenting the most intense peak in the UV-vis spectrum. In this study it was found that temperatures above 80 °C during AgNP synthesis caused precipitation in the reaction mixture, leading to NP instability. This was be attributed to the thermal degradation of the extract organic material at higher temperatures, particularly the breakdown of proteins, which are known to play a crucial role in the green synthesis process [126].

These studies highlight the significant impact of temperature on the green synthesis of MNPs, influencing their size, stability, and overall properties. Depending on the synthesis conditions and the biological extract used, temperatures may influence NP yield and stability. Generally, temperatures close to 100 °C are commonly employed in green synthesis protocols to promote efficient reduction and nucleation of metal ions [127]. However, optimizing temperature parameters is crucial to tailoring NP size, shape and functionality for specific applications.

#### 4.3.2. Influence of pH

In addition to temperature, pH can also influence the morphology, and the particle size of the MNPs synthesize through green processes, as previously described by Parial et al. in the synthesis of AuNPs [117]. Similarly to temperature, the influence of pH in the MNPs characteristics varies within the different studies.

In a study by Velgosová et al., the extract of the microalgae *Parachlorella kessleri* was used to synthesize AgNPs at different pH, including 2, 4, 6, 8 and 10. UV-vis spectroscopy confirmed successful AgNP synthesis at all tested pH values except for pH 2. TEM analysis revealed that AgNPs formed at pH 4 exhibited a larger particle size (20–60 nm), when compared to those synthesized at pH 10, which had an average size of 15 nm. Additionally, AgNPs synthesized at pH values below 6 were found to lose stability over time. The study concluded that pH 8 was optimal for AgNP synthesis [128].

Similarly, Costa et al. synthesized AuNPs using a brown macroalgae extract, *Sargassum cymosum*, at different pH values, (2–3, 7, 7.5 and 12). UV-vis spectroscopy indicated that AuNPs synthesized at pH 2–3 exhibited the highest peak, suggesting a more efficient synthesis process. DLS analysis further demonstrated that as the pH decreased from 12 to 7.5, the particle size decreased while the PdI increased. However, the optimal pH range for the synthesis was reported to be between 3 and 5, where stable NPs formation was observed without inhibiting the reaction [129].

Overall, lower pH values, such as pH 2, have been reported to minimize nucleation and promote aggregation of MNPs. In contrast, moderately alkaline pH values, around pH 8–9, can help prevent NPs aggregation, thereby enhancing stability [130]. However, excessively high pH values may inhibit the reduction reaction, limiting the formation of MNPs [129]. Furthermore, pH can influence the structure and functionality of the algae bioactive molecules, which can result in a change in their ability to interact and reduce the metal precursor [131]. Therefore, maintaining a controlled pH environment is essential for achieving desirable NPs characteristics through green methods. 

#### 4.3.3. Metal Precursor Concentration

The concentration of the metal precursor in green synthesis also significantly influences the synthesis of MNPs, affecting particle size, morphology, and reaction time.

In a study by Rahman et al., the microalgae *Chlamydomonas reinhardtii*, a wild type and a cell wall deficient strain, were used for the intracellular synthesis of AgNPs. AgNO_3_ was tested at different concentrations of 1.250 mmol/L and 0.625 mmol/L. The formation of AgNPs was confirmed by a color change in the reaction mixture, from green to dark brown. UV-vis spectroscopy confirmed that the highest concentrations of AgNO3, for both strains, exhibited the most intense peaks, suggesting greater NPs formation. However, at lower AgNO_3_ concentrations, the reaction proceeded more rapidly. TEM analysis revealed that higher AgNO_3_ concentrations resulted in larger AgNPs, although all synthesized NPs remained below 10 nm in size [132].

Variations in precursor concentration can significantly influence the rate of nucleation, particle size, and yield. In general, higher concentrations promote increased NP formation; however, they may also lead to larger particle sizes or agglomeration if stabilizing agents are insufficient [133]. These findings demonstrate that metal precursor concentration plays a crucial role in NP synthesis, directly impacting reaction kinetics and final particle characteristics.

#### 4.3.4. Algae Species and Concentration

The species and concentration of algae used in the green synthesis of MNPs significantly influence the characteristics of the resulting NPs [38].

Aboelfetoh et al. used a green macroalgae, *Caulerpa racemosa*, to formulate AgNPs, using different extract concentrations (1, 3 and 5 mL). The NPs were analyzed by surface plasmon resonance (SPR) which revealed that as the extract concentration increased, the SPR band exhibited a blue shift and greater intensity, indicating a reduction in particle size and an increase in AgNP formation efficiency [134].

The effect of algae species on NP synthesis was further examined by Nagarajan et al., who evaluated three algae *Caulerpa peltata*, *Hypnea valencia* and *Sargassum myriocystum*, for their ability to produce ZnNPs. UV-vis spectroscopy confirmed that only *Sargassum myriocystum* exhibited a characteristic ZnNP peak, demonstrating its capability for NP synthesis. Further studies on extract concentration revealed that a lower extract volume (5 mL) was optimal, whereas higher concentrations (>25 mL) led to NP aggregation. Additionally, morphological analysis showed that higher extract concentrations resulted in ZnNPs with triangular, hexagonal, and rod-like shapes, while lower extract concentrations favored the formation of spherical ZnNPs [135].

The type of algae, as well as the concentration of their bioactive compounds used in green synthesis, significantly influences the characteristics of the resulting MNPs. As previously discussed, various bioactive compounds are responsible for both the reduction of metal ions and the stabilization of the NPs. Consequently, differences in algal species lead to differences in NP morphology, size, and stability, due to the distinct biochemical profiles of each species. Similarly, the concentration of bioactive compounds plays a critical role in NP formation. Higher concentrations typically increase nucleation rates, leading to a faster synthesis process. However, this can result in smaller NPs due to rapid and simultaneous nucleation events [127]. These findings emphasize the critical role of algae species selection and extract concentration in determining NP size, shape, and stability.

### 4.4. Application of Metallic Nanoparticles from Marine Sources

The green synthesis of MNPs from natural sources is recognized as a cost-effective and environmentally friendly approach, offering a sustainable alternative to conventional chemical and physical synthesis methods. The ability to control NP properties through the aforementioned mentioned synthesis parameters has expanded their applicability across various industries. Notably, MNPs synthesized via green methods have shown immense potential in biomedical and pharmaceutical fields, where their biocompatibility, stability, and functional properties contribute to advancements in drug delivery, diagnostics, and therapeutic applications [47,52].

#### 4.4.1. Gold Nanoparticles (AuNPs)

AuNPs have been extensively researched due to their unique properties, including their electrical, optical, and photothermal characteristics, as well as their biocompatibility and versatility in functionalizing a wide range of ligands [136,137,138]. These attributes have made AuNPs highly valuable in a broad range of medical applications, particularly in drug delivery, diagnostics, imaging, and the therapy sector [53].

The electrical properties of AuNPs, which enable them to conduct electricity and respond to external electric fields, make them useful for diagnostic devices [139]. For example, AuNPs can serve as signal amplifiers, enhancing the electrical response for the detection of biomolecules or pathogens. This makes them suitable for diagnosing diseases such as cancer, cardiovascular conditions, and infectious diseases [140]. Furthermore, AuNPs’ electrical properties can be applied for the development of neural interfaces to monitor brain activity and stimulation of neural tissues, which is crucial for treating neurological disorders like Parkinson’s disease, epilepsy, and other neurodegenerative conditions [141].

The optical properties of AuNPs further enhance their utility in the medical field. Their ability to interact with light, particularly in the visible and near-infrared regions, enables their use in various imaging and diagnostic techniques [139]. For example, AuNPs are utilized in surface plasmon resonance (SPR)-based sensors, which detect biomarkers or pathogens with high sensitivity, making them useful for early disease detection [140].

Additionally, the optical and photothermal properties of AuNPs have therapeutic applications, such as in photothermal therapy (PTT), where AuNPs absorb light and convert it into localized heat. This heat selectively destroys cancer cells while minimizing damage to surrounding healthy tissues, making PTT a promising approach for targeted cancer treatment [142].

AuNPs are also highly versatile in drug delivery applications due to their ability to function with various ligands. This capability enables precise targeting of specific tissues or cells, enhancing the efficacy and accuracy of treatments, particularly in precision medicine. Furthermore, the surface charge of AuNPs can optimize drug delivery by facilitating cellular uptake, as NPs with a positive charge interact more readily with negatively charged cell membranes [139]. AuNPs also exhibit antimicrobial properties, making them effective in treating bacterial and fungal infections. This broadens their application to include the treatment of infectious diseases [143].

While traditional synthesis methods for AuNPs often rely on harsh chemicals such as cetyltrimethylammonium bromide (CTAB) or citrate, which can produce toxic by-products, there has been a growing interest in green synthesis methods. These methods, which utilize natural products like algae, retain the discussed properties and additionally exhibit biocompatibility. As a result, AuNPs synthesized through green methods, particularly those derived from marine organisms, have emerged as ideal candidates for various biomedical applications (Table 1) [47,138].

#### 4.4.2. Silver Nanoparticles (AgNPs)

Among the diverse range of MNPs, AgNPs stand out as one of the most widely studied and commercialized MNPs [47]. These NPs have various attributes, including electrical, thermal, optical, and biological properties [151]. As a result, AgNPs tend to be preferred over AuNPs, and are widely used across many industries, including the food and pharmaceutical industry, as well as in drug delivery and even in agriculture [152].

Silver has one of the highest levels of electrical conductivity when compared to other metals. As a result, AgNPs have remarkable electrical properties, presenting significant potential to be used as biosensors or in electronic devices that require electrical conductivity [153].

The surface of AgNPs can be modified, which is an excellent characteristic that enhances their effectiveness in applications like drug delivery and bioimaging, as it improves the safety and bioavailability of the biological molecules or other materials [153]. For example, in cancer therapy, AgNPs have been used effectively as nanocarriers of chemotherapeutic agents, since they allow better control over the tumor and can potentially decrease some of the side effects, when compared to the chemotherapeutic agent alone [154].

In recent years, silver has gained significant attention due to its antimicrobial potential, since it has been successfully tested against various pathogenic microbes [155]. The exact mechanism behind the antimicrobial properties of silver is still not entirely known, but it is believed that the interaction between the AgNPs and microbial cell membranes causes substantial damage to the membranes, resulting in significant toxicological damage [109].

When compared to AuNPs, AgNPs tend to be more biodegradable, which is particularly important for applications in medicine and environmental remediation. The biodegradability of AgNPs reduces the risk of long-term toxicity or accumulation in biological systems, a concern that is more pronounced with AuNPs [156].

Similarly to AuNPs, the traditional methods used to synthesize AgNPs have disadvantages, such as the toxicity of the reagents used, namely sodium citrate or sodium borohydride [157]. Therefore, the incorporation of algae in the green synthesis of these NPs is not only more environmentally friendly but also has the potential to add beneficial effects to AgNPs, thus providing them with improved medicinal properties [109]. Moreover, these AgNPs have demonstrated high stability, solubility and yield, making them highly promising for various applications. Accordingly, the green synthesis of AgNPs with marine algae is viewed as a simple and rapid method, associated with many potential applications, such as anticancer and antimicrobial therapies (Table 2) [53,151].

#### 4.4.3. Other Metallic Nanoparticles (MNPs)

While AuNPs and AgNPs have gained widespread use in the medical field due to their unique properties, several other MNPs based on metallic oxides are being actively explored for medical applications. The interest in these alternative NPs comes from their specific advantages over gold and silver, such as lower cost, reduced toxicity, magnetic properties, or other functional characteristics [166,167].

MNPs such as copper (CuNPs), and cobalt nanoparticles (CoNPs) present various advantages, making them viable for large-scale applications. For instance, CuNPs are more cost-effective when compared to AuNPs or AgNPs [168]. CuNPs and CoNPs can also be controlled with an external magnetic field, enabling targeted drug delivery and enhanced imaging. This magnetic behavior offers a distinct advantage over AuNPs and AgNPs, which lack such properties. CoNPs can also serve as contrast agents in magnetic resonance imaging (MRI), providing high-resolution, non-invasive diagnostic techniques [169,170]. CuNPs are also gaining interest in their antimicrobial properties, making them effective against bacteria, fungi, and viruses [171].

Others MNPs, such as zinc oxide (ZnO) are considered to be non-toxic, being ideal to be applied in medicine. ZnO NPs (ZnONPs) also exhibit strong antibacterial, antifungal, and antiviral properties, being commonly used in wound healing, in controlling infections, as well as being used as food preservatives [172].

Currently, there are a few studies based on the green synthesis of MNPs such as zinc, copper and cobalt, using marine-derived algae and other natural sources (Table 3). The use of metal oxides has been increasing, as they are able to produce NPs with varying morphologies and other useful properties [109]. The use of algae in NP synthesis offers several benefits, including reduced toxicity and environmental impact [52]. As research continues, it is expected that more MNPs, including zinc, copper, and cobalt, will be integrated into medical treatments, providing more sustainable and cost-effective alternatives to AuNPs and AgNPs.

### 4.5. Challenges and Limitations of Green Synthesis Using Marine Sources

While the green synthesis of MNPs using marine sources, such as algae, offers numerous advantages, including eco-friendliness and reduced toxicity, it also presents several challenges and limitations that need to be addressed for practical applications and large-scale applications.

As previously discussed, various parameters, such as temperature, pH, metal precursor concentration, among many others, influence the synthesis process. Even small changes in these factors can lead to significant differences in the MNPs’ characteristics, such as particle size, morphology, and surface properties [179]. Therefore, precise control of these parameters is essential to ensure the desired properties of the MNPs and consistency.

Another significant limitation is the limited long-term stability of the synthesized MNPs, which compromises the storage, transportation and application of these NPs. Both the yield and stability of MNPs can fluctuate depending on the selected synthesis conditions and the type of marine organism used.

Although various bioactive compounds have been shown to be able to reduce and stabilize the MNPs, the exact mechanisms and molecular pathways involved are not fully understood. This lack of information complicates the optimization of the synthesis protocols.

Choosing the appropriate algae species for MNP production can be difficult, as the properties of the resulting NPs can vary significantly, not only between species but also across different growth stages or algae concentrations [38]. Furthermore, the concentration and composition of bioactive compounds within the same species can fluctuate due to environmental factors such as seasonal and climatic changes, reducing reproducibility [55]. This variability presents a significant obstacle that must be addressed before this green synthesis method can be scaled for widespread use [180].

In most synthesis protocols, algae extracts, rather than isolated compounds, are used. These extracts contain complex mixtures of bioactive molecules, which may interact synergistically or antagonistically during the reduction, nucleation, and growth phases. These interactions may significantly affect the physicochemical properties of the MNPs, including their size, shape, surface charge, stability, and functional behavior [181].

Additionally, some algae, particularly from groups like *Rhodophyta*, *Chlorophyta*, and *Pyrrhophyta* groups, can produce natural toxins. Even at low concentrations, the presence of these toxins can pose environmental and human health risks if not properly controlled [182]. Therefore, careful monitoring and regulation are necessary when sourcing marine organisms for green NP synthesis to ensure both environmental and human safety. Also, the use of certain metal precursors may introduce toxicity, in the intracellular synthesis methods this may adversely affect the algae viability, lowering NP yield or damaging the biomass [183].

While the use of marine algae for the green synthesis of MNPs offers sustainability and cost advantages, several ecological and practical impact limitations may interfere with a large-scale implementation and economic feasibility. Overharvesting of marine organisms, such as algae, could lead to environmental imbalances or depletion of species, especially if the demand for green-synthesized NPs grows. For instance, excessive removal of algae can disrupt coastal ecosystems, affecting nutrient cycling, marine biodiversity, and habitat structure. If not properly regulated, this expanding interest may also place unsustainable pressure on other marine species and fragile habitats. To minimize ecological impact, sustainable sourcing practices and responsible cultivation methods for marine organisms should be prioritized.

One promising solution is the large-scale cultivation of algae through aquaculture, particularly algae farming. This approach reduces reliance on wild populations, ensures a more consistent biomass supply, and can even provide environmental benefits, such as carbon sequestration and nutrient removal from surrounding waters [184]. Economic feasibility of using marine sources for NP synthesis can be impacted by factors such as seasonal variability, large-scale cultivation costs, and the availability of specific algae species. The ecological impact of harvesting marine organisms and the potential for contamination in coastal environments also needs to be carefully evaluated, especially when considering the scalability of marine-derived MNPs for industrial applications or biomedical applications.

These considerations highlight the importance of conducting comprehensive economic assessments to ensure the responsible and sustainable use of marine resources in NP synthesis. Despite the growing interest in marine-based NP production, assessments of sustainability and scalability remain limited. Life cycle assessment (LCA) of nanomaterials and techno-economic analyses data are still insufficient or lacking in critical data. Several efforts have been made to overcome this concern. An example is a 2021 review of 71 LCA studies conducted between 2001 and 2020 found that only five addressed exposure pathways of nanomaterials, and 92% did not include uncertainty analysis [185]. The authors emphasized the need for more detailed and transparent life cycle inventory data, as well as the importance of considering environmental impacts and potential risks of nanomaterials, including those specific to marine sourced nanomaterials.

In summary, although the green synthesis of MNPs from marine algae presents significant potential, it also faces several technical, ecological, and economic challenges. Further research is required to optimize the synthesis process, including selecting the sustainable algae species, refining the synthesis conditions and evaluating the environmental and economic viability of large-scale production. Addressing these limitations will be crucial for advancing the green synthesis of marine-derived NPs and enabling its widespread use.

## 5. Polysaccharide-Based Nanoparticles from Marine Sources

Similarly to MNPs, polysaccharide-based NPs have attracted significant attention due to their excellent biocompatibility, biodegradability and versatility for functionalization. Polysaccharides derived from marine sources, such as crustaceans, marine algae, and microorganisms, are among the most abundant and sustainable natural biopolymers [29,186]. Furthermore, these compounds are widely available, structurally stable, and cost-effective, making them highly attractive for a broad range of applications, particularly in the biomedical and pharmaceutical sectors [29].

Marine polysaccharides exhibit a wide range of biofunctional properties, which are largely determined by their structural composition and functional groups. In the last decades, these polysaccharides have been extensively investigated for their potential applications [187].

### 5.1. Fucoidan

**Figure 3:**
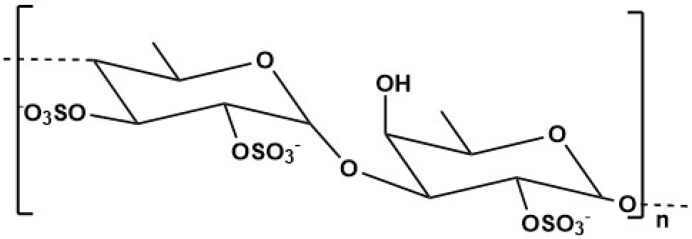
Fucoidan structure.

Fucoidan, a natural sulphated polysaccharide, can be extracted from many sources, including marine invertebrates, with brown algae, such as *Fucus vesiculosus*, *Undaria pinnatitinda* and *Ecklonia kurome*, considered to be the major sources. This compound is composed of xylose, L-fucose, mannose and galactose (Figure 3) [188,189]. Fucoidan is a negatively charged polysaccharide, with high biocompatibility and biodegradability, and low toxicity. For example, in the United States, fucoidan extracted from *Fucus vesiculosus* and *Undaria pinnatifida* was approved and recognized as a food ingredient by the Food and Drug Administration (FDA) [190].

In a study by James et al., fucoidan was extracted from *Ascophyllum nodosum*, a brown macroalgae. In this study, four extraction methods were selected; namely, microwave, subcritical water extraction, thermochemical extraction and ultrasonication, using glycerol and choline chloride as solvents. The results demonstrated that the microwave-assisted extraction method presented the highest yield, with a value of 22.24 wt% [191].

In another study by Tang et al., another brown macroalgae, *Kjellmaniella crassifolia*, was selected to extract fucoidan. The extraction was based on enzymes, including β-glucosidase and cellulase, and a subsequent multi-step ethanol precipitation procedure. This method of extraction resulted in a fucoidan yield of 4.74% [192].

Fucoidan has been widely applied in the medical and pharmaceutical sector. Boo et al. reported that fucoidan from *Undaria pinnatifida* induce apoptosis in PC-3 prostate cancer cell by activating ERK1/2 MAPK and the inactivation p38 MAPK apoptosis [193]. Similarly, Takahashi et al. verified that fucoidan from *Cladosiphon novae-caledoniae* Kylin was able to reduce levels pro-inflammatory cytokines, including IL-1β, IL-6, and TNF-α, in cancer patients, suggesting anti-inflammatory and immunomodulatory effects [194].

### 5.2. Chitosan

**Figure 4:**
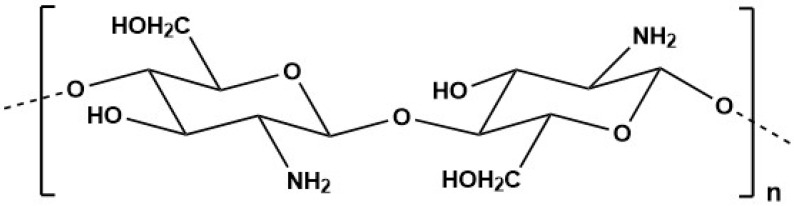
Chitosan structure.

Marine-derived chitosan (Figure 4), a cationic polymer derived from chitin (the primary component of crustacean’s exoskeletons), can be found in the shells of some marine microorganisms, including crustaceans. Chitosan is typically produced through the N-deacetylation of chitin, which is one of the most abundant natural biopolymers on Earth. This polysaccharide is typically found in insect cuticles, the shells of crabs and shrimps, as well as in the cell walls of fungi, yeasts and green algae [195,196].

Varma et al. extracted chitosan from *Modiolus modiolus*, known as horse mussel, using sequential steps of decolorization, demineralization, and deproteinization, obtaining a chitin yield of 40.13%. This chitin was then subjected to N-deacetylation, producing chitosan, with a yield of 10.21% [197]. Similarly, in a study by Varun et al., shrimp shell waste was used to extract both chitin and chitosan. Chitin was first isolated using a demineralization and deproteinization method, resulting in a 14.72% yield. This was followed by N-deacetylation, chitosan was extracted with a yield of 12.03% [198].

This polysaccharide has also been applied in cancer therapy. Li et al. concluded that this chitosan enhanced the anti-tumor activity of natural killer cells, as well as promoted their survival, supporting its potential in cancer treatment [199]. In cardiovascular treatment, Jiang et al. evaluated the use of chitosan to improved antioxidant capacity and decrease lipids profiles in patients with coronary heart disease, indicating a cardioprotective effect [200].

### 5.3. Alginate

**Figure 5:**
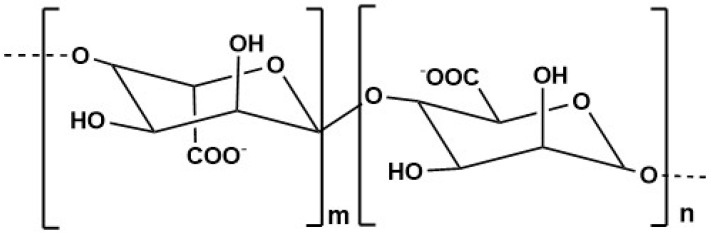
Alginate structure.

Alginate (Figure 5) is a marine-derived polysaccharide that has gained significant attention due to its remarkable physicochemical properties. As a naturally occurring anionic polymer, alginate offers several advantages, including low toxicity, excellent biocompatibility, and cost-effectiveness [201]. These characteristics have made alginate a promising candidate for a wide range of medical applications having approval from the FDA for use in human health products [202]. This polymer can be found in the cell walls of brown seaweeds such as *Ascophyllum nodosum*, *Laminaria digitata*, *Laminaria japonica* and *Macrocystis pyrifera* [201,202].

One common method for alginate extraction involves sequential acidic and alkaline treatments of brown seaweed. For example, Nøkling-Eide et al. extracted alginate from two brown algae, *Alaria esculenta* and *Saccharina latissima*. Saccharina latissima reporting an optimized condition. The study determined that optimum conditions for extraction are a short extraction time and an alkaline treatment at pH of 9. With this extraction process, it was possible to obtain yields of 229 ± 12 and 185 ± 7 mg/g dry weight seaweed, for *Alaria esculenta* and *Saccharina latissima*, respectively [203]. Similarly, Bertagnolli et al., extracted alginate from *Sargassum filipendula* using formaldehyde and hydrochloric acid treatment, obtaining a yield of 17.2% [204]. Alginate demonstrates excellent medical properties; Lukova et al. reported that alginate derived from *Ericaria crinita* significantly reduced serum levels of pro-inflammatory cytokines TNF-α, IL-1β, and IL-6 in a rat model of LPS-induced systemic inflammation, indicating strong antioxidant and anti-inflammatory potential [205]. Supporting this, Acevedo et al. found that alginate oligosaccharides improved the viability of gastric cells under oxidative stress by reducing intracellular ROS levels, and activated the Nrf2 pathway, a key regulator of cellular defense mechanisms [206].

### 5.4. Synthesis of Polysaccharide-Based Nanoparticles

Marine polysaccharides are often used for the development of DDS, due to their biocompatibility and biodegradability [207]. Among marine polysaccharides, fucoidan, chitosan and alginate are the most studied ones for NP applications, due to their unique properties and versatility [29,202].

The synthesis of polysaccharide-based NPs, similar to the methods of MNPs, can generally be classified into top–down and bottom–up approaches. Common techniques used in their production include ionic gelation and self-assembly, typically classified as physical methods, as well as chemical methods such as emulsification, cross-linking, and polyelectrolyte complexation [208]. Among these, ionic gelation has gained significant interest due to its simplicity and versatility in controlling particle size and morphology [209].

Ionic gelation is a physical process that involves the formation of NPs by the electrostatic interaction between a charged polysaccharide and counterions, typically in an aqueous medium [210]. For instance, Bavel et al. used this method to formulate chitosan NPs, which was prepared by adding, drop by drop, tripolyphosphate, an anionic cross linker, into a chitosan solution into tripolyphosphate. This method proved to be very promising and reproducible. These NPs were seen to interact with negatively charged proteins and DNA [211].

These synthesis methods are applied for the formulation of fucoidan, chitosan and alginate NPs. Recently, due to their complementary properties, chitosan–fucoidan hybrid NPs are being developed to enhance stability, drug loading, and controlled release, making them highly promising for biomedical applications. Given their chemical properties, where fucoidan has a negative charge and chitosan a positive one, electrostatic interactions between the two polysaccharides can occur, with this process resulting in the formation of polyelectrolyte complexes, by self-assembly [18,212].

One promising method to formulate this type of NPs is via the ultrasonification method, in which chitosan and fucoidan are mixed at various mass ratios and sonicated at room temperature. This method was previously applied by Huang et al. to evaluate the potential of chitosan–fucoidan NPs as oral drug delivery systems. In this study, the NPs were evaluated at various pH conditions to mimic the gastrointestinal environment. The study found that increasing pH results in increased particles, and the NPs exhibited a spherical shape. Additionally, when the NPs were loaded with curcumin, their release was suppressed at lower pH values but facilitated at higher pH values, which demonstrate the potential of these type of NPs as DDSs [213].

Another method to synthesize these types of NPs is the dropping method, which consists of adding, drop by drop, a fucoidan solution to a chitosan solution under stirring [18]. In a study by Huang et al., chitosan–fucoidan NPs were formulated following this protocol, using a modified chitosan derivate (O-Carboxymethyl chitosan). In this study, curcumin was encapsulated in the NPs, and effective cellular uptake of curcumin was observed in a colon adenocarcinoma cell line (Caco-2) [214].

When comparing NPs obtained through the ultrasonification and those obtained via the dropping method, the first method resulted in NPs with an average particle size between 200 and 300 nm, whereas the dropping method resulted in smaller NPs, with an average size of 100 and 200 nm. Studies by Huang et al. highlighted the potential of using chitosan-fucoidan NPs for oral delivery, showing both effective compound release and enhanced cellular uptake [213,214].

The yield of chitosan–fucoidan NPs produced per unit weight of biomass can vary significantly depending on several factors, including the ratio of chitosan to fucoidan, extraction and synthesis methods used, the purity of the polysaccharide used, and other reaction conditions. For example, a study by Lee et al. investigated the effect of the pH level of chitosan solution and the chitosan–fucoidan mass ratio on NP formation. The yield, calculated gravimetrically, showed that pH 5 and 1:1 chitosan–fucoidan mass ratio were suggested as ad hoc optimum conditions to prepare chitosan–fucoidan NPs for high yield, small size and good suspension stability [215].

### 5.5. Application of Polysaccharide-Based Nanoparticles from Marine Sources

Polysaccharide-based NPs are highly versatile and sustainable materials, widely used across various fields due to their biodegradability, biocompatibility, and eco-friendly nature. In the biomedical and pharmaceutical sectors, these NPs have been extensively studied and principally being applied as DDS [29,210].

#### 5.5.1. Fucoidan Nanoparticles

The development of fucoidan-based NPs has attracted considerable attention, since research has demonstrated that the biological activities of fucoidan are improved when used as a nanocarrier. Fucoidan-based NPs recently demonstrated a huge impact in the medical field, showing strong anti-coagulant and antiviral properties [216].

In a study by Wardani et al., fucoidan-based NPs were formulated using the high-energy ball milling method, which ground the polysaccharide powder into particles. These NPs were formulated with an average particle size of 267.2 nm and administrated to diabetic rats (Wister rats). The study observed that the NPs reduced oxidative stress in aortic endothelial cells. Specifically, it was observed that the levels of reactive oxygen species (ROS) decreased following the administration of 300 mg of NPs/kg body weight. It was also shown that at the same concentration, levels of antioxidant enzymes super dismutase (SOD) and glutathione peroxidase (GPx), increased when compared to untreated rats. The study concluded that the NPs had the potential to prevent vasoconstriction of the aortic lumen by increasing its diameter [217].

Chiang et al. formulated fucoidan NPs from *Fucus vesiculosus* using an emulsification method, producing NPs with an average size of 216.3 nm. These NPs demonstrated anticancer properties, as they induced a reduction in cell viability in metastatic breast adenocarcinoma (MDA-MB-231). Furthermore, when administered intravenously to ICR mice, the NPs reduced tumor volume by 2.49-fold compared to the control group, highlighting their potential as an effective anticancer agent [218].

Fucoidan NPs have also been explored for drug delivery applications. A study reported by Lee et al. that also used fucoidan from *Fucus vesiculosus*, formulated acetylated fucoidan NPs with an average size of 137.5 nm using a dialysis method, and was able to highly efficiently encapsulate chemotherapeutic agent doxorubicin (71.1%). In macrophage cells (Raw264.7) treated with the NPs, the expression of pro-inflammatory cytokines TNF-α and GM-CSF, increased by 1.13 and 1.86-fold, respectively. Additionally, in a colorectal adenocarcinoma cell line (HCT-8), doxorubicin internalization after two hours was approximately 99% when encapsulated in NPs compared to only 1.79% for the free drug. These findings demonstrate that fucoidan-based NPs significantly enhance the delivery efficiency of doxorubicin to cancer cells [219].

#### 5.5.2. Chitosan Nanoparticles

Recently, the formulation of chitosan-based NPs has received significant interest, as these types of NPs demonstrated the ability to release drugs in a slow and controlled manner. Furthermore, these types of NPs can improve the solubility and stability of drugs, thus decreasing their toxicity, and making them ideal for use as delivery systems [220]. Chitosan NPs are positively charged, which enhances adhesion, useful for drug delivery.

In a study by Razi et al., chitosan NPs were formulated with a coacervation process and were encapsulated with interleukin-12 (IL-12). These NPs, with an average particle size of 381.8 nm, were tested in a fibrosarcoma mouse model (BALB/c). The results demonstrated that the NPs were able to enhance the expression of IL-12 and IFN-γ, as well as decrease the tumor volume [221].

Similarly, in a study performed by Chen et al., chitosan NPs were formulated with an ionic cross-linking method and had an average size inferior to 200 nm. These NPs were used to encapsulate IR780 iodide and/or 5-Aminolevulinic acid (5-ALA) agents. The encapsulation efficiency of NPs containing only IR780 was 76.3%, whereas for the NPs with both IR780 and 5-ALA, the encapsulation efficiency was 83.0% and 34.6%, respectively. The stability of the NPs was confirmed at different pH levels (5.5–7.4). When orally administered to BALB/c mice, followed by laser treatment at 808 nm (photothermal therapy) and 635 nm (photodynamic therapy), the results showed that tumor growth was effectively suppressed in mice treated with both laser irradiation and dual-loaded NPs [222].

#### 5.5.3. Chitosan–Fucoidan Nanoparticles

Chitosan and fucoidan are among the most extensively studied polysaccharides. Due to their complementary properties, chitosan–fucoidan hybrid NPs are being developed to enhance stability, drug loading, and controlled release, making them highly promising for biomedical applications. Therefore, chitosan–fucoidan NPs have significant potential to be applied in many sectors, with the medical field being a very promising area of study in the future (Table 4).

#### 5.5.4. Alginate Nanoparticles

Given its excellent properties, alginate has been widely used to formulate NPs. These alginate-based NPs have attracted significant interest, as a result of their exceptional biocompatibility, as well as their excel capacity for controlled drug release [202,230].

In a study by Roque et al., alginate NPs were formulated for oral treatment against *Candida albicans*. These NPs were formulated with an average particle size of 886.7 nm and were used to encapsulate an antifungal agent nystatin. The study reported an encapsulation efficiency of 71.78% and demonstrated that nystatin was released from the NPs within two hours. Additionally, encapsulated nystatin exhibited a larger inhibitory zone against *C. albicans* compared to free nystatin, indicating improved antifungal efficacy [231].

Similarly, Urzedo et al. formulated alginate-based NPs encapsulating S-nitroso-mercaptosuccinic acid, with a particle size of 271.1 nm and an encapsulation efficiency of 98.7%. These NPs were combined with AgNPs and tested against *E. coli*, *S. aureus*, and *Streptococcus mutans*, demonstrating a good anti-bacterial activity [232].

### 5.6. Challenges and Limitations in Polysaccharide-Based Nanoparticles

In recent years, polysaccharide-based nanoparticles have attracted considerable attention given their excellent biocompatibility, biodegradability, and promising potential in various applications, particularly as DDS [186]. Despite the extensive research and promising developments in the formulation and application of these NPs, several limitations and challenges remain, which prevent their scalability and consistency in real-world applications [233].

One of the main challenges regarding polysaccharide NPs is not related to the synthesis, but in the extraction of the polysaccharides. Some water-based extraction methods, which are based on environmentally friendly methodologies, typically have a low extraction efficiency and require extended time. Other extraction techniques using other type of solvents, though more efficient, often involve toxic chemicals that are not environmentally friendly and therefore necessitate careful removal prior to use [234].

The purification of polysaccharides after extraction is also another major challenge. Multiple purification steps are often required, as extracts often contain a mixture of proteins, lipids, and other contaminants [234,235]. Even when standardized extraction and purification protocols are applied, batch-to-batch variations may still occur. These variations can lead to differences in molecular weight, chain length, and degree of polymerization of the extracted polysaccharides, affecting the reproducibility and functionality of the final polysaccharide-based NPs [236].

These occurrences complicate the ability to obtain a large-scale industrial application and regulatory approval. Consequently, further research is required for more efficient, scalable and standardized methodologies for extraction and purification.

Additionally, when using marine polysaccharides of crustacean origin, such as chitosan, in biomedical applications, there is a potential risk of immunotoxicity in individuals allergic to shellfish [237].

In conclusion, while the field of polysaccharide-based nanoparticles continues to advance and shows immense promise, addressing these limitations is crucial. Continued innovation and interdisciplinary research are needed to develop scalable, safe, and effective nanoparticle systems that meet the rigorous demands of modern biomedical or other applications.

## 6. Conclusions

The ocean is an extraordinary and largely unexplored ecosystem, home to most of the living organisms, which are highly diverse. As our understanding of marine organisms increases, so does the demand for its bioactive compounds, driven by their unique and valuable biological properties. The exploitation of marine bioactive compounds for the formulation and incorporation of NPs represents an innovative and rapidly evolving field in nanotechnology and biomedical research. This review has highlighted the potential of marine resources, including macroalgae, microalgae and crustaceans, as sources of bioactive molecules that can improve the synthesis, stability and functionality of NPs.

In recent years, the green synthesis of MNPs has gained significant attention as a sustainable and eco-friendly approach. The application of marine bioactive compounds in the formulation of marine-derived MNPs has demonstrated tremendous potential, especially in the biomedical and pharmaceutical sector. However, limitations persist including the lack of mechanistic understanding underlying their synthesis, variability in the marine biomass that can influence the efficacy and properties of the NPs produced and challenges in achieving reproducibility and scalability. Therefore, further research is essential to fully understand the role of synthesis parameters and biomolecules to optimize and improve consistency in NP size, shape and biofunctionality, with the aim of unlocking their full potential.

Marine polysaccharides, namely fucoidan, chitosan and alginate, have demonstrated significant potential for the development of DDS, due to their biological properties, including biocompatibility, biodegradability, and their ability to effectively control drug release. However, extraction and purification of marine polysaccharides is a major limitation for reproducibility and scalability, particularly due to batch-to-batch variability and, additionally, for biomedical applications the risks of immunotoxicity in shellfish-allergic individuals. Furthermore, the majority of research has focused only on some specific polysaccharides, while others of marine origin, such as carrageenan, remain largely underexplored, but are promising for future applications in DDS formulations.

As research in this area continues to grow, the unique combination of growth in marine biological resource exploration and nanotechnology is expected to result in the development of sustainable and effective NPs for future biomedical and pharmaceutical applications. To ensure clinical success and widespread use of these NPs, it will be crucial for future studies to focus on standardized, economically viable and environmentally friendly methods. Equally important is the integration of comprehensive sustainability assessments, such as LCA and techno-economic analysis to guide the scalable and sustainable commercialization of marine-based NPs. Addressing these challenges through interdisciplinary collaboration and responsible innovation will be essential to enable the transition of marine nanotechnology from laboratory-scale research to real-word application.

## Figures and Tables

**Table 1:** Particle size, morphology and biomedical applications of gold nanoparticles (AuNPs) synthesized via marine algae, as reported in multiple studies.

Type of Algae		Particle Size (nm)	Morphology	Activities (Tested)	References
Microalgae	*Chlorella sorokiniana*	20–40	Spherical	Antifungal (*Candida tropicalis*, *C. glabrata* and *C. albicans*)	[144]
	*Dunaliella salina*	22.4	Spherical	Anticancer (MCF-7 cell line)	[145]
Macroalgae	*Chondrus crispus*	16.9	Spherical	Anti-inflammatory (THP-1 cell line)	[146]
	*Codium tomentosum*	34.5	Spherical	Anticancer (HepG2 and BxPC-3 cell lines)	[147]
	*Gelidium corneum*	15.0	Spherical	Anti-inflammatory (THP-1 cell line)	[146]
	*Porphyra linearis*	44.2	Spherical	Anti-inflammatory (THP-1 cell line)	[146]
	*Sargassum myriocystum*	15.0	Spherical, triangular	Cardiovascular Treatment	[148]
	*Stoechospermum marginatum*	18.7–93.7	Spherical, triangular, hexagonal	Antibacterial (*Pseudomonas aeruginosa*, *K. oxytoca*, *Enterobacter faecalis*, *K. pneumoniae*, *Vibrio parahaemolyticus*, *V. cholerae*, *Salmonella typhii*, *S. paratyphi* and *P. vulgaris*)	[149]
	*Undaria pinnatifida*	6.8	Spherical	Antimicrobial (*Escherichia coli*, *S. aureus*, *P. aeruginosa*, *C. albicans* and *C. auris*)	[150]

**Table 2:** Particle size, morphology and biomedical applications of silver nanoparticles (AgNPs) synthesized via marine algae, as reported in multiple studies.

Type of Algae		Particle Size (nm)	Morphology	Activities (Tested)	References
Microalgae	*Chlorella minutissima*	73.13	Spherical	Antibacterial (*Bacillus cereus*, *S. aureus*, *E. coli*, *Klebsiella* sp. and *Salmonella* sp.)	[158]
	*Chlorella vulgaris*	11.52	Spherical	Antimicrobial (*Bacillus* sp., *Erwinia* sp. and *Candida* sp.)	[159]
	*Spirulina platensis*	2.23–14.68	Spherical	Anticancer (A549, HCT and Hep2 cell lines)	[160]
	*Spirogyra varians*	17.6	Spherical	Antibacterial (*S. aureus*, *B. cereus*, *Salmonella typhimurium*, *E. coli*, *Listeria monocytogenes*, *P. aeruginosa* and *Klebsiella* sp.)	[161]
Macroalgae	*Acanthophora specifera*	33–81	Cubic	Antimicrobial (*S. aureus*, *B. subtillis*, *Salmonella* sp., *E. coli* and *C. albicans*)	[162]
	*Gelidiella sp.*	40–50	Spherical	Anticancer (HepG2 cell line)	[163]
	*Sargassum vulgare*	6.90–16.97	Spherical	Anticancer (HepG-2, HCT-116, HeLa and PC-3 cell lines) Antibacterial (*S. caprae*, *S. capitis* and *S. epidermidis*)	[164]
	*Turbinaria turbinata*	14.50–39.85	Spherical	Antifungal (*Botrytis cinerea*, *Rhodotorula mucilaginosa*, *Penicillium expansum*, *Alternaria alternate* and *Stemphylium vesicarium*)	[165]

**Table 3:** Particle size, morphology and biomedical applications of other metallic nanoparticles synthesized via marine algae, as reported in multiple studies.

Type of Algae		Types of NPs	Particle Size (nm)	Morphology	Activities (Tested)	References
Microalgae	*Arthrospira platensis*	Zinc oxide	30.0–55.0	Spherical	Antimicrobial (*S. aureus*, *B. subtilis*, *E. coli*, *P. aeruginosa* and *C. albicans*)	[173]
	*Chlorella vulgaris*	Zinc oxide	28.2	Hexagonal	Anticancer (PC12 cell line)	[174]
	*Chlorella vulgaris*	Zinc oxide	21	Rod	Antibacterial (*E. coli*, *P. aeruginosa* and *S. aureus*)	[175]
	*Spirulina platensis*	Cobalt oxide	3.52	Flake	Antifungal (*C*. *albicans*, *C*. *glabrata* and *C. krusei*)	[176]
	*Spirulina platensis*	Copper oxide	3.75–12.4	Spherical	Anticancer (A549, HCT and Hep2 cell lines)	[160]
Macroalgae	*Bifurcaria bifurcata*	Copper oxide	5.0–45.0	Spherical	Antibacterial (*E. aerogenes* and *S. aureuso*)	[177]
	*Gracilaria edulis*	Zinc oxide	66–95	Rod	Anticancer (PC-3 cell line)	[178]

**Table 4:** Particle size, morphology and biomedical applications of chitosan-fucoidan nanoparticles, as reported in multiple studies.

Particle Size (nm)	Morphology	Application	References
190–230	Quasi-spherical	Radio Therapy	[223]
300.0–400.0	Quasi-spherical	Oral Drug Delivery (Quercetin)	[224]
130–150	Quasi-spherical	Intranasal Drug Delivery (Curcumin) Anti-inflammatory (ICR mice)	[225]
160	Spherical	Drug Delivery (Gemcitabine) Anticancer (SKBR3 cell line and NSG mice)	[226]
380	Spherical	Oral Drug Delivery	[227]
230–250	Spherical	Pulmonary Delivery (Gentamicin)	[228]
300	Irregular	Oral Drug Delivery (Methotrexate) Anticancer (A549 cell line)	[229]
